# Mechanisms of Racial and Ethnic Differences in Cognitive Aging

**DOI:** 10.1093/geroni/igaa057.2824

**Published:** 2020-12-16

**Authors:** Andrea Rosso, Jennifer Manly

## Abstract

Racial and ethnic disparities in age-related cognitive function and dementia risk in the US are well recognized. However, the psychosocial drivers of these disparities and underlying mechanisms are less well studied. This symposium will highlight novel research regarding our current understanding of racial/ethnic differences in brain and cognitive aging and the underlying mechanisms of the disparities. Frist, two papers will describe results regarding racial/ethnic differences in cognitive function and brain aging markers. Few studies have assessed racial/ethnic differences in cognitive function across age groups. Indira Turney will utilize data from a multigenerational study to explore how age impacts racial/ethnic differences in cognitive function. Underlying brain mechanisms of racial/ethnic differences in cognitive outcomes are also not well defined. Sara Godina will present a systematic review of racial/ethnic differences in structural markers of brain aging and neuropathology. Second, three papers will explore how various risk factors may explain the racial/ethnic disparities in cognitive outcomes. Melissa Lamar will demonstrate the differential associations of various blood pressure indicators with cognitive change among black older adults. B. Gwen Windham will present data from two studies that illustrate differential effects of common risk factors by race and region, highlighting inherent difficulties in race-place disparity research. Finally, Laura Zahodne will present results on how psychosocial factors, beyond socioeconomic status, contribute to racial/ethnic disparities in cognitive function. Jen Manly will lead a discussion on the implications of these results for the future of dementia prevention efforts for an increasingly diverse older US population.

